# Simple, scalable and robust purification of two HIV-1 subtype C gp120 monomer subunit antigens for phase II clinical trial in Republic of South Africa

**DOI:** 10.1186/1742-4690-9-S2-P356

**Published:** 2012-09-13

**Authors:** Y Wen, S Stephenson, C Zambonelli, S Hilt, M Wininger, A Dey, S Barnett, A Carfi

**Affiliations:** 1 Novartis, Cambridge, MA, USA

## Background

Development of an effective vaccine against HIV-1 is challenging due to various viral evolutionary mechanisms to evade human immune system. The partial efficacy of the recent RV144 vaccine efficacy trial in Thailand provides hope for improvements of vaccine regimens for higher efficacy. A Phase IIb proof-of-concept clinical trial in the Republic of South Africa (RSA) is planned to confirm and extend the results of the RV144 trial with the vaccine strategy of poxvirus vector prime plus envelope protein boost.

## Methods

We selected two HIV subtype C gp120 vaccine antigens, TV1.C gp120 and 1086.C gp120, formulated with Novartis proprietary adjuvant, MF59 as protein boosts of the clinical trial.

## Results

To produce TV1.C gp120 and 1086.C gp120 monomers, we generated CHO stable cell lines for both gp120, which consistently expressed gp120 subunits with high yield. Simple, scalable and robust antigen purification processes were developed to generate both gp120 proteins. The ion-exchange based purification strategy enabled the separation of gp120 monomer from dimer and produced gp120 monomer with high purity and homogeneity.

## Conclusion

Purified gp120 monomers were stable, either alone or in combination, and when formulated with adjuvant MF59. Finally, the early evaluations showed that both gp120 monomers were immunogenic and able to elicit high neutralizing antibody titer.

